# Inhibition of Gastric Acid Secretion by H2 Receptor Antagonists Associates a Definite Risk of Enteric Peritonitis and Infectious Mortality in Patients Treated with Peritoneal Dialysis

**DOI:** 10.1371/journal.pone.0148806

**Published:** 2016-02-12

**Authors:** Miguel Pérez-Fontan, Daniela Machado Lopes, Alba García Enríquez, Beatriz López-Calviño, Andrés López-Muñiz, Teresa García Falcón, Ana Rodríguez-Carmona

**Affiliations:** 1 Division of Nephrology, University Hospital A Coruña, A Coruña, Spain; 2 Division of Nephrology, Hospital de Gaia, Vilanova de Gaia, Portugal; 3 Division of Epidemiology^2^, University Hospital A Coruña, A Coruña, Spain; Hospital Universitario de La Princesa, SPAIN

## Abstract

**Background:**

Evidences linking treatment with inhibitors of gastric acid secretion (IGAS) and an increased risk of serious infections are inconclusive, both in the population at large and in the particular case of patients with chronic kidney disease. We have undertaken an investigation to disclose associations between treatment with IGAS and infectious outcomes, in patients undergoing chronic Peritoneal Dialysis (PD).

**Method:**

Observational, historic cohort, single center design. Six hundred and ninety-one patients incident on PD were scrutinized for an association among treatment with IGAS (H2 antagonists H2A or proton pump inhibitors PPI) (main study variable), on one side, and the risks of enteric peritoneal infection (main outcome), overall peritoneal infection, and general and infectious mortality (secondary outcomes). We applied a three-step multivariate approach, based on classic Cox models (baseline variables), time-dependent analyses and, when appropriate, competing risk analyses.

**Main results:**

The clinical characteristics of patients treated with H2A, PPI or none of these were significantly different. Multivariate analyses disclosed a consistently increased risk of enteric peritonitis in patients treated with IGAS (RR 1.65, 95% CI 1.08–2.55, p = 0.018, Cox). Stratified analysis indicated that patients treated with H2A, rather than those on PPI, supported the burden of this risk. Similar findings applied for the risk of infectious mortality. On the contrary, we were not able to detect any association among the study variables, on one side, and the general risks of peritonitis or mortality, on the other.

**Conclusions:**

Treatment with IGAS associates increased incidences of enteric peritonitis and infectious mortality, among patients on chronic PD. The association is clear in the case of H2A but less consistent in the case of PPI. Our results support the convenience of preferring PPI to H2A, for gastric acid inhibition in PD patients.

## Introduction

Inhibitors of gastric acid secretion (IGAS) are widely prescribed for prevention and management of upper gastrointestinal tract disease, including gastroesophageal reflux, gastritis and peptic ulcer. Treatment with this family of drugs has been associated with many side effects, from minor manifestations (diarrhea, headache, flatulence…) to more consequential complications, including hypersensitivity reactions, nutritional deficits, bone marrow suppression, bone fractures, neurotoxicity, hepatotoxicty and gastric tumors [1]. However, the significance of some of these associations is questionable and, as a whole, IGAS are viewed as relatively safe drugs.

Several recent reports have raised concerns about a potential risk of serious infections among individuals treated with any of the two main groups of IGAS, namely H2 receptor antagonists (H2A) and proton pump inhibitors (PPI). Pulmonary [2,3] and enteric infections, including *Clostridium difficile* enterocolitis [4–6], could be particularly frequent, in these patients. The mechanisms underlying this apparent predisposition are not totally clear, but colonization of the upper gastrointestinal tract by enteric bacteria, disruption of the natural competence of the intestinal barrier, overgrowth of multirresistant bacteria or drug-induced disorders affecting the bactericidal capacity of leukocytes have all been quoted as potential explanations [5,7].

Patients with chronic kidney disease (CKD) are frequently treated with IGAS, due to the high prevalence of gastrointestinal symptoms and disorders, which may be present in as much as 70% of these individuals [8]. The incidence of upper gastrointestinal bleeding is also markedly increased, in this setting [9]. The reasons underlying this predisposition are complex, including the uremic milieu itself, comorbidity and polipharmacy, among other factors. The association between treatment with IGAS and the risk of infection in patients with CKD has been insufficiently investigated. In the particular case of patients undergoing chronic peritoneal dialysis (PD), there is a specific concern that treatment with these drugs could promote peritoneal infections by enteric bacteria, but the available studies are relatively small, suffer significant methodologic limitations and have provided controversial results. We have undertaken a better powered approach to this question, applying multivariate strategies of analysis, to control for expected imbalances among patients, regarding treatment with IGAS.

## Method

### General design

Following a longitudinal, historic cohort design, we investigated the association between treatment with IGAS (main study variable) and selected outcomes of a relatively large sample of patients starting PD in a reference, university medical center during the period January 1995—December 2013. Follow-up was closed by March 2015. The main outcome variable was the risk of peritoneal infection by enteric bacteria (estimated as survival to first episode). Secondary outcome variables included the overall risk of peritoneal infection, and the risks of general and infectious mortality. We performed general analyses for the use of IGAS, and also in separate for PPI and H2A. We applied univariate and multivariate strategies of analysis, including time-dependent strategies and, when appropriate, a competing risk approach.

This study complied with the requirements of the local ethic committee of the University Hospital of A Coruña (Spain) for retrospective, observational studies. Data were fully anonymized for their management. Given the retrospective design of the study, neither written or oral informed consent was requested form participant patients.

### Study population

The study population included all patients starting PD in our centre between January 1995 and December 2013 (follow-up closed by March 2015), under the following inclusion criteria:

- Age >10 years
- Minimum follow-up on PD of two months
- Clinical records available
- Information on treatment with IGAS available during follow-up

IGAS were generally prescribed for management of dyspepsia, prevention of gastroesophageal disease (gastritis, peptic ulcer) or treatment of gastroesophageal reflux.

### Study variables

The main study variable was treatment with IGAS, either managed as a binary variable or in separate for PPI and H2A. We did not contemplate either the doses of IGAS prescribed or the different drugs in each group. The overwhelming majority of patients were treated with the PPI omeprazole or the H2A ranitidine.

The main outcome variable was the risk of peritoneal infection caused by enteric bacteria (*Enterobacteriaceae*, *Enterococcus spp*. and/or enteric anaerobes), including polimicrobial infections. We excluded cases with an overt surgical etiology. Secondary outcome variables included the general risks of peritoneal infection and overall and infectious mortality.

Control variables included demographic data (age, gender), body mass index (Weight/Height^2^), comorbidities [diabetes, Charlson’s comorbidity score, malnutrition (assessed by standard subjective global assessment SGA, and managed as a binary variable), previous or current immunosuppressive treatment and, specifically, background of previous atherothrombotic events (coronary heart disease, thromboembolic stroke, aortic aneurism and/or peripheral vascular disease demanding intervention or hospital admission)] and congestive heart failure, laboratory variables [plasma albumin (autoanalyzer), hemoglobin (autonalyzer), C-reactive protein (immunoturbidimetry)] and composite estimations, including glomerular filtration rate GFR (mean of urea and creatinine renal clearances) and peritoneal transport characteristics (D/P creatinine at 240’ during peritoneal equilibration test).

The use of ISAG increased progressively during the study period (40.1% of patients starting PD 1995–2004 vs 64.8% of those starting 2005–2013). Moreover, more patients starting PD before 2005 used H2A. For this reason, PD vintage was maintained as a control variable in multivariate models, despite the fact that it did not perform as an independent predictor of any of the outcome variables.

### Data analysis

Basic comparisons were produced according to Student’s t test, one-way ANOVA, Mann-Whitney’s test and χ^2^ distribution analyses. We applied multivariate stepwise logistic regression analysis to disclose the main demographic, clinical and biochemical correlates of treatment with IGAS. Univariate survival analyses for the main and secondary outcomes were performed according to Kaplan Meier plots (log rank). Patients were censored in cases of loss to follow-up, kidney transplant, drop-out to hemodialysis, death (peritoneal infection) or non-infectious death (infectious mortality). Missing data were managed by pairwise deletion. Baseline C-reactive protein values were available in only 560 patients (81.0%); but all the other baseline control variables could be recorded in more than 97% of patients. Given the expected imbalances among patients on different IGAS treatments, we also performed multivariate analyses, aimed at disclosing the adjusted risks for each outcome. We carried out this in three steps

1. We first performed stepwise Cox analyses to investigate the association among baseline treatment with PPI and H2A, on one side, and the outcome variables, on the other. Patients were censored in the above mentioned cases. Only first-degree interactions were explored for final models.
2. In a second step, we performed time-dependent Cox analyses, to take into account expected variations in treatment with PPI and H2A and other variables, during follow-up. We followed Murphy [10] and Fisher [11] models, for this purpose.
3. As a final step, we performed Fine and Grey competing risks analyses, to correct for the potential distorting effects of mortality (overall) or non-infectious mortality on the association among treatment with PPI/H2A, on one side, and the main outcome variables, on the other. In these models, overall mortality was managed as a competing risk for peritoneal infection (in case of peritonitis-related mortality the infection was recorded as an event previous to the demise), while non-infectious mortality was managed as a competing risk for infectious mortality. Plots of accumulated incidences were produced according to Kalbefleisch and Prentice.

We used the SPSS and Stata V10 softwares for data analysis.

## Results

**Fig 1:**
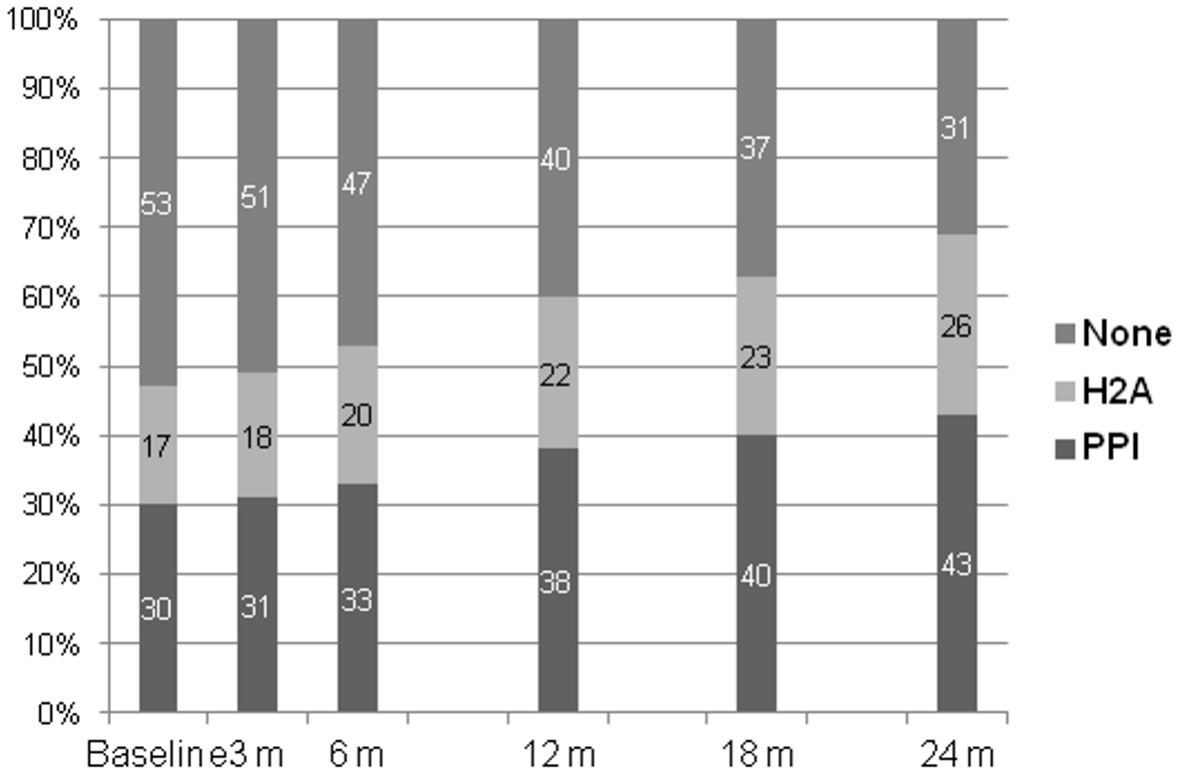
Proportion of patients treated with different types of inhibitors of gastric acid secretion during the first two years of follow-up on PD (p<0.0005).

Six hundred and ninety-one patients fulfilled the inclusion criteria and were considered for analysis. The demographic, clinical and laboratory characteristics of the patients showed significant differences, according to baseline treatment with IGAS (Table 1). Eight patients (3 on H2A, 3 on PPI and 2 on none of the previous) presented advanced liver disease at the start of PD. Multivariate, logistic regression analysis identified lower plasma albumin (odds ratio OR 0.93, 95% CI 0.90–0.96, p<0.0005), former or ongoing immunosuppressive therapy (OR 4.18, 95% CI 2.15–8.15, p<0.0005), a background of atherothrombotic events (OR 2.09, 95% CI 1.44–3.00, p<0.0005), older age (OR 1.02, 95% CI 1.01–1.03, p = 0.003), higher hemoglobin levels (OR 1.12, 95% CI 1.01–1.25, p = 0.036), malnutrition (OR 1.76, 95% CI 1.13–2.75, p = 0.013) and PD started 2005–2013 (OR 2.78, 95% CI 1.63–4.44, p<0.0005) as independent correlates of treatment with IGAS (any type) at the initiation of PD. On the other hand, lower plasma albumin (OR 0.95, 95% CI 0.90–0.99, p = 0.028), former or ongoing immunosuppressive therapy (OR 3.25, 95% CI 1.32–7.80, p<0.0005) and PD started 2005–2013 (OR 4.58, 95% CI 1.84–9.36, p<0.0005) were the main factors independently associated with prescription of PPI rather than H2A, among patients treated with IGAS. On the other hand, the use of these drugs tended to increase during follow-up on PD, from 47% of patients, at baseline, to 69% at the end of the second year (Fig 1).

**Table 1:** Baseline characteristics of the study population.

(N)	PPI (207)	H2A (119)	None (366)	p
Vintage (PD before 2005)(%)	64 (31.1)	87 (73.5)	255 (69.9)	0.0005
Age (years)	58.8 (16.0)	64.2 (13.7)*	57.4 (15.7)	0.0005
Gender (% males)	63.6	55.9	59.6	0.38
Diabetes (%)	85 (41.1)	44 (31.3)	117 (32.0)	0.40
Charlson’s score	4.4 (2.2)	4.4 (2.2)	3.6 (1.9)*	0.0005
Previous atherothrombotic event (%)	84 (40.6)	59 (50.0)	113 (30.9)	0.0005
Previous congestive heart failure (%)	54 (26.1)	36 (30.5)	49 (13.4)	0.0005
Modality of PD (% APD)	67 (32.4)	57 (48.3)*	135 (36.9)	0.016
Previous/Ongoing immunosuppression (%)	38 (18.4)*	8 (6.8)	16 (4.4)	0.0005
Malnutrition (%)	25 (12.1)	17 (14.4)*	25 (6.8)	0.009
GFR (mL/m)	6.7 (4.0)*	5.5 (3.7)	5.7 (3.6)	0.007
Body mass index (Kg/m^2^)	25.9 (4.7)	26.1 (4.7)	25.8 (4.4)	0.81
Plasma albumin (g/L)	35.5 (6.1)	36.3 (5.3)	38.1 (5.4)*	0.0005
Hemoglobin (g/dL)	10.7 (1.6)	10.6 (1.6)	10.3 (1.6)	0.055
C-reactive protein (mg/dL)	0.64(0.10, 52.4)	0.89 (0.10, 19.8)	0.53 (0.10, 16.7)**	0.016
D/P 240’ creatinine	0.67 (0.13)	0.66 (0.13)	0.65 (0.14)	0.39

PPI: Proton pump inhibitors; H2A: H2 receptor antagonists; APD: Automated PD; GFR: Glomerular filtration rate

Figures denote mean values (SD) for numerical variables, median (range) for C-reactive protein and n (%) for categorical variables. Comparisons by ANOVA (Scheffé), χ^2^ distribution and Mann Whitney’s test.

* Significant difference vs any other group.

** Significant difference vs H2A

**Fig 2:**
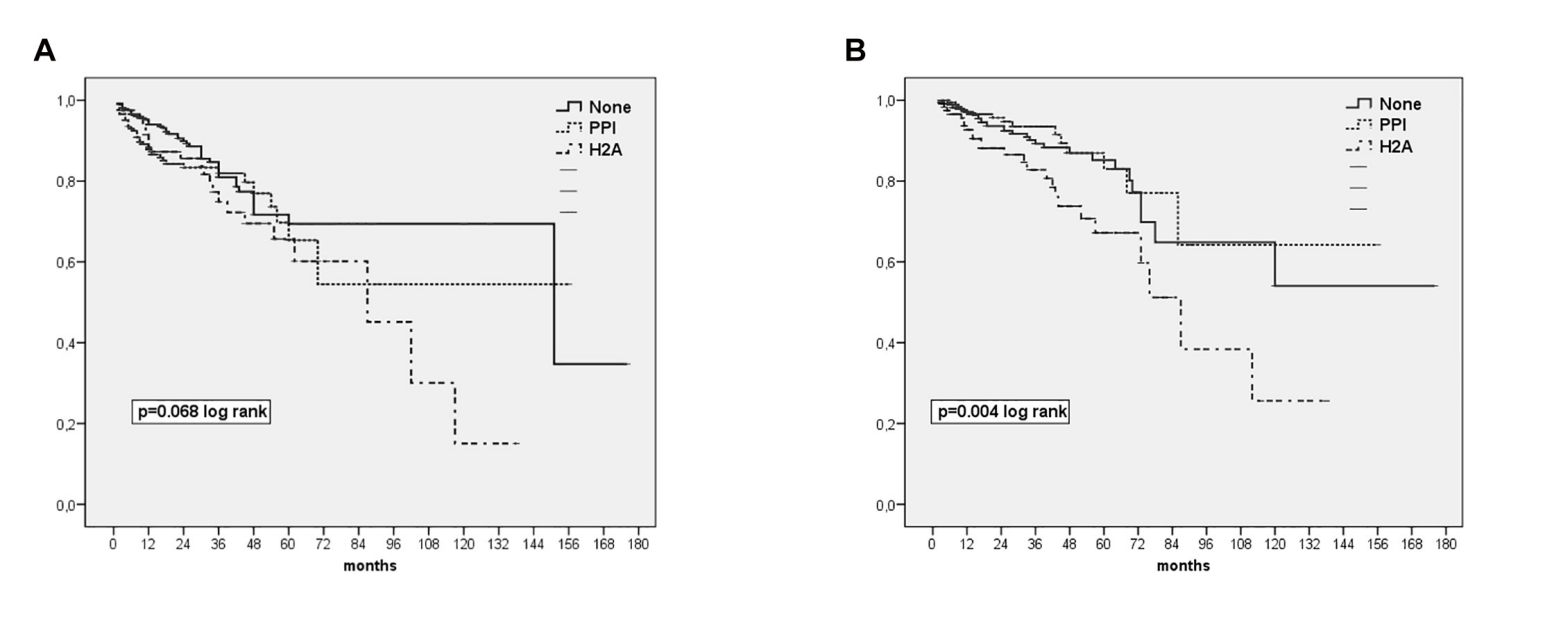
**(a)**. Survival to first episode of enteric peritonitis according to baseline treatment with IGAS (b) Survival to infectious mortality according to baseline treatment with IGAS.

Table 2 displays the main events and outcomes during follow-up (28.9 ± 24.4 months). Only 15 patients (2.2%) were lost to follow-up due to transfer to another center. The global incidence of peritonitis was 1 episode every 32.2 patient-months for patients on PPI at baseline, as compared with 1/33.4 for patients on H2A and 1/36.2 for patients not treated with IGAS (p = 0.33). Univariate (Kaplan-Meier) analyses displayed trends to an increased risk of enteric peritonitis (Fig 2a) and infectious mortality (Fig 2b) for patients treated with H2A (but not PPI) at the initiation of PD. On the contrary, no such association was observed for the overall risks of peritonitis (p = 0.22, log rank) or mortality (p = 0.18).

**Table 2:** Main outcomes according to baseline treatment with inhibitors of gastric acid secretion.

	PPI (n = 207)	H2A (n = 118)	None (n = 366)
**Death**	86 (41.5)	73 (61.9)	159 (43.4)
Cardiovascular	45 (21.7)	30 (25.4)	76 (20.8)
Infectious	17 (8.2)	25 (21.2)	33 (9.0)
Failure to thrive-PD suspended	10 (4.8)	8 (6.8)	14 (3.8)
Neoplasia	3 (1.4)	1 (0.8)	10 (2.7)
Other	11 (5.3)	9 (7.6)	26 (7.1)
**Causes of infectious mortality**			
Peritoneal infection	8	17	19
Pulmonary infection	3	1	11
Bacterial endocarditis	1	0	0
Urinary tract infection	0	2	0
Biliary tract infection	0	2	0
Septicaemia (other/unknown origin)	3	4	1
Other	1	0	0
**Peritonitis (patients suffering at least one episode)**	117 (56.5)	70 (59.3)	191 (52.2)
**Peritonitis (number of episodes)**	181 (100)	108 (100)	289 (100)
Grampositives	87 (48.1)	52 (48.1)	143 (49.5)
Gramnegatives	35 (19.3)	26 (24.1)	47 (16.3)
Polimicrobial	26 (14.4)	13 (12.0)	45 (15.6)
Fungal (primary)	6 (3.3)	5 (4.6)	13 (4.5)
Mycobacteria	0	0	1 (0.3)
Negative culture	27 (14.9)	12 (11.1)	40 (13.8)
**Enteric peritonitis (≥ 1 episode)**	41 (19.8)	30 (25.4)	61 (16.7)

PPI: Proton pump inhibitors; H2A: H2 receptor antagonists

Figures denote number of patients (% versus total number of patients) except for the etiologic agents of peritonitis (number of episodes)

Table 3 presents the results of multivariate analysis for the main outcome variables, according to baseline variables. Patients on IGAS presented a definitely increased risk of enteric peritonitis during follow-up. Stratified analysis revealed that this risk was overt only for patients on H2A, while the trend for patients on PPI did not reach statistical significance. Infectious mortality was increased only in patients on H2A therapy, while we did not observe any association among treatment with IGAS, on one side, and general mortality or overall risk of peritonitis, on the other. Neither did we detect any significant interaction among treatment with IGAS, on one side, and control variables, on the other.

**Table 3:** Impact of baseline treatment with inhibitors of gastric acid secretion on study outcomes. Multivariate

	HR	95% CI	p	Control variables
**Enteric peritonitis**				Age, Immunosuppression, GFR, C reactive protein
IGAS overall	1.65	1.08, 2.55	0.018	
PPI	1.61	0.98, 2.51	0.059	
H2A	1.67	1.02, 2.80	0.042	
**Infectious mortality**				Age, diabetes, Charlson, albumin, Immunossuppresion, GFR
IGAS overall	1.09	0.65, 1.82	0.75	
PPI	0.68	0.35, 1.32	0.26	
H2A	1.78	1.01, 3.21	0.049	
**Overall peritonitis**				Age, albumin, immunosuppression, GFR, modality of PD
IGAS overall	1.18	0.95, 1.47	0.11	
PPI	1.23	0.96, 1.59	0.10	
H2A	1.08	0.80, 1.47	0.61	
**Overall mortality**				Age, diabetes, Charlson, albumin, immunossuppresion, GFR
IGAS overall	0.96	0.76, 1.23	0.76	
PPI	0.85	0.64, 1.13	0.26	
H2A	1.15	0.85, 1.55	0.37	

IGAS: Inhibitors of gastric acid secretion; PPI: Proton pump inhibitors; H2A: H2 receptor antagonists.

* Independent predictors of outcome in best model. PD vintage NS

Stepwise Cox models. Figures denote adjusted hazard ratios (HR) with 95% confidence intervals and p values for the main outcome variables.

Table 4 displays the results of multivariate, time-dependent analyses. Overall, the results were quite similar to those observed for baseline variables although, in this case, the specific risk of enteric peritonitis for patients on H2A did not reach statistical significance.

**Table 4:** Impact of treatment with inhibitors of gastric acid secretion on study outcomes. Time dependent, multivariate analysis.

	HR	95% CI	p	Control variables
**Enteric peritonitis**				Diabetes, Charlson, albumin, immunosuppression, hemoglobin, GFR
IGAS overall	1.33	1.02, 1.75	0.040	
PPI	1.08	0.70, 1.66	0.74	
H2A	1.45	0.92, 2.29	0.11	
**Infectious mortality**				Age, diabetes, Charlson, albumin, immunosuppression, hemoglobin, GFR
IGAS overall	1.61	1.14, 2.27	0.007	
PPI	0.90	0.52, 1.49	0.62	
H2A	2.03	1.19, 3.47	0.01	
**Overall peritonitis**				Age, albumin, immunosuppression, GFR, hemoglobin, modality of PD
IGAS overall	1.09	0.95, 1.60	0.24	
PPI	1.32	0.95, 1.66	0.17	
H2A	0.97	0.74, 1.26	0.80	
**Overall mortality**				Age, diabetes, Charlson, albumin, immunosuppression, hemoglobin, GFR
IGAS overall	1.11	0.93, 1.31	0.24	
PPI	0.94	0.76, 1.29	0.94	
H2A	1.16	0.88, 1.53	0.31	

IGAS: Inhibitors of gastric acid secretion; PPI: Proton pump inhibitors; H2A: H2 receptor antagonists.

* Independent predictors of outcome in best model. PD vintage NS

Time dependent Cox’s models. Figures denote adjusted hazard ratios (HR) with 95% confidence intervals and p values for the main outcome variables.

**Fig 3:**
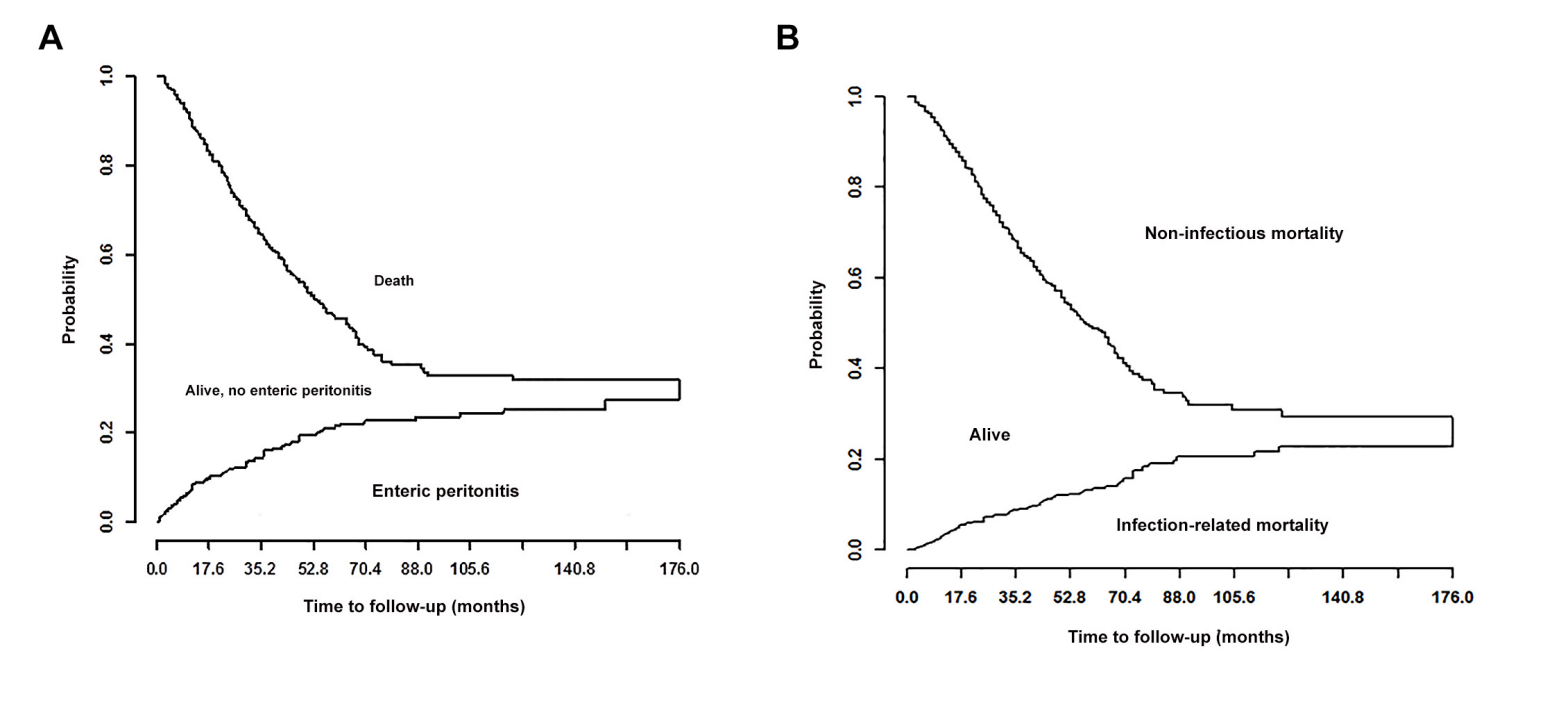
**(a)**. Competing risk plot for enteric peritonitis) competing event, death for any cause) (b) Competing risk plot for infectious mortality (competing event death for noninfectious causes)

Fig 3a and 3b display Kalbefleisch-Prentice plots of competing risks for enteric peritonitis and infectious mortality, respectively. Table 5 shows the results of competing risk analysis for the main outcome variables, with trends similar to the other two multivariate approaches. Treatment with IGAS associated a trend to an increased risk of enteric peritonitis, and a definite risk of infectious mortality, particularly in patients on H2A therapy.

**Table 5:** Impact of treatment with inhibitors of gastric acid secretion on study outcomes. Competing risks analysis.

	HR	95% CI	p	Control variables*
**Enteric peritonitis**				Diabetes, Charlson, albumin, immunosuppression, hemoglobin, GFR
IGAS overall	1.51	1.00, 2.28	0.052	
PPI	1.11	0.79, 2.65	0,62	
H2A	1.40	0.90, 2.18	0.14	
**Infectious mortality**				Age, diabetes, Charlson, albumin, immunosuppression, hemoglobin, GFR
IGAS overall	1.35	0.98, 1.88	0.081	
PPI	0.58	0.32, 1.02	0.066	
H2A	2.25	1.36, 3.70	0.0015	
**Overall peritonitis**				Age. albumin, immunosuppression, GFR, hemoglobin, Modality of PD
IGAS overall	1.20	0.97, 1.51	0.09	
PPI	1.20	0.95, 1.52	0.12	
H2A	1.04	0.80, 1.35	0.76	

IGAS: Inhibitors of gastric acid secretion; PPI: Proton pump inhibitors; H2A: H2 receptor antagonists.

* Independent predictors of outcome in best model. PD vintage NS

Competing events: Overall mortality (overall and enteric peritonitis) and non-infectious mortality (Infectious mortality)

## Discussion

Our results are consistent with the hypothesis that patients on PD who are treated with IGAS present increased risks for enteric peritonitis and infectious mortality. There are several potential explanations for these associations. First, sustained inhibition of gastric acid secretion alters the bacterial ecology of the gastrointestinal tract, not just favoring gastric colonization by enteric bacteria [12], but also modifying the flora of the lower gut [13]. Secondly, both hypochlorhydria and bacterial overgrowth may facilitate microbial translocation across the intestinal barrier. Thirdly, experimental studies have suggested that suppression of gastric acid production may selectively promote colonization of the large intestine by pathogenic, multirresistant bacteria [14]. Finally, IGAS may have detrimental effects on the bactericidal capacity of neutrophils [15]. The potential clinical consequences could be increased incidences of enteric infections [4], including *Clostridium difficile* enterocolitis, and pulmonary infections [7]. Some specific subsets of patients could sustain a particularly high risk of suffering these complications, including cirrhotics with ascites [16,17] and hospitalized patients on antibiotic therapy [6]. CKD may also represent a high risk setting, because the intestinal barrier and the microbiota of these patients are frequently altered [18], and treatment with IGAS could further complicate these disorders.

PD may represent a particularly high risk setting for treatment with IGAS, due to the specific risk of peritoneal infections in these patients. Few studies have investigated the infectious risk associated with IGAS among patients on PD. Caravaca et al [19] presented the results of an observational survey on a group of 55 patients treated with PD, with the aim of disclosing predictors of enteric peritonitis. Logistic regression analysis identified treatment with either H2A or PPI as the only consistent predictor of this complication. On the contrary, del Peso et al [20] were unable to detect such association in a sample of a similar size (n = 57). In 2008, Nessim et al [21] presented the results of a case-control analysis of 228 episodes of peritonitis in 134 PD patients, comparing factors associated with enteric versus non-enteric peritonitis. Treatment with IGAS did not make any difference among groups, although stratified analyses disclosed a trends to an association between H2A therapy and infections by enteric microbia. A more recent case-control analysis on 120 patients [22] suggested an increased incidence of peritonitis (overall), but not of enteric peritonitis, among patients treated with H2A.

The results of our study appear to support previous reports [21,22], suggesting that any association between treatment with IGAS and peritoneal infections in PD patients may be largely restricted to patients treated with H2A, while the evidence linking treatment with PPI and these infections was much less consistent. At first sight, this difference is difficult to explain, because both types of IGAS appear to carry similar side effects predisposing to infection. It is possible that treatment with H2A may associate unidentified, additional risks not present in the case of PPI. For instance, Kwon et al [22] have suggested that the prolonged half-life of H2A, but not of PPI, in the presence of CKD [23] could promote some of the negative effects of these drugs, in this setting.

This study has significant limitations. The observational, nonrandomized design does not permit to establish a causality link between treatment with IGAS and outcomes. Moreover, the significant differences among patients, according to treatment with these drugs (Table 1), may raise the possibility that IGAS may just be a confounding factor for other, unknown variables. The fact that the specific indications for starting IGAS were not clear in many cases may add further uncertainty. To correct for these potential biases, we applied three different multivariate strategies of analysis, controlling for all the main variables related to outcomes, obtaining similar results. Remarkably, we observed no association among treatment with IGAS, on one side, and the general risks of peritonitis or mortality, on the other, suggesting a specific association to infectious events related to gastrointestinal bacteria. On the other hand, our study provides the best powered evidence on this question published to date, permitting effective multivariate approaches to data analyses, and providing consistent answers to the main questions under consideration.

In summary, treatment with IGAS associates increased incidences of enteric peritonitis and infectious mortality among patients treated with chronic PD. This association is clear in the case of H2A, but much less consistent in the case of PPI. Our results support the convenience of preferring PPI to H2A for gastric acid inhibition or, at least, to use lower doses of H2A, in these patients. Only a randomized study may be able to establish a causal link among these factors.
